# The relation between natural variations in ocean heat uptake and global mean surface temperature anomalies in CMIP5

**DOI:** 10.1038/s41598-018-25342-7

**Published:** 2018-05-09

**Authors:** Sybren Drijfhout

**Affiliations:** 1 University of Southampton, Southampton, UK; 2 Royal Netherlands Meteorological Institute, De Bilt, The Netherlands

## Abstract

It is still unclear whether a hiatus period arises due to a vertical redistribution of ocean heat content (OHC) without changing ocean heat uptake (OHU), or whether the increasing radiative forcing is associated with an increase in OHU when global mean surface temperature (GMST) rise stalls. By isolating natural variability from forced trends and performing a more precise lead-lag analysis, we show that in climate models TOA radiation and OHU do anti-correlate with natural variations in GMST, when GMST leads or when they coincide, but the correlation changes sign when OHU leads. Surface latent and sensible heat fluxes always force GMST-variations, whilst net surface longwave and solar radiation fluxes have a damping effect, implying that natural GMST-variations are caused by oceanic heat redistribution. In the models an important trigger for a hiatus period on decadal timescales is increased reflection of solar radiation, by increased sea-ice cover over deep-water formation areas. On inter-annual timescales, reflection of solar radiation in the tropics by increased cloud cover associated with La Niña is most important and the subsequent reduction in latent heat release becomes the dominant cause for a hiatus.

## Introduction

Earth’s average surface air temperature (SAT) has risen more than 1 degree since the late 19^th^ century, but the trend in GMST was particularly low in the period 1998–2012^1^, leading to speculation at the time that the forcing had diminished. This period has been called a surface warming hiatus^2^ or warming-trend slowdown^3,4^. Reduced radiative forcing due to a weakening of greenhouse gases or solar forcing, or due to an increase in stratospheric water vapour or anthropogenic aerosols can indeed cause a hiatus period. An inventory of energy changes in the climate system^5–7^, however, does not support a large role for these processes, in which case the energy imbalance at the TOA, being the result of a positive greenhouse gas forcing, should have been reduced, which is not observed. As a result, it was concluded that ocean warming had continued unabated during the hiatus period^8,9^, and the hiatus was explained as a surface temperature phenomenon that did not represent a slowdown in warming of the planet, but rather a vertical redistribution of heat within the oceans^4,10^. Some studies argued that increased heat transport to deeper layers occurred^11,12^, while others favoured a more shallow redistribution of the heat between the Pacific and Indian Oceans^13,14^. To what extent heat was sequestered in the deeper ocean and by which processes is still debated^4,15^.

## The forcing-feedback-response model

The question whether, apart from a vertical redistribution of OHC, more heat is sequestered in the deep ocean in a hiatus period is important, giving its implications for the energy balance of the planet. The Earth’s response to changes in radiative forcing can be explained by a simple energy balance model^16^. This model predicts that a reduced energy imbalance arises when external forcing causes a hiatus, while an increased imbalance arises when it is due to internal climate dynamics. The forced response is described by

1 $${\rm{Q}}={\rm{\lambda }}\,{\rm{\Delta }}{\rm{T}}+{\rm{N}}$$

where Q is the net radiative forcing, λ ΔT is the change in net radiation due to a temperature change ΔT, λ is the climate feedback parameter and N is the TOA net downward radiation, which equals OHU. In this simple model OHU is related to ΔT through ocean heat uptake efficiency, κ:

2 $${\rm{N}}={\rm{\kappa }}\,{\rm{\Delta }}{\rm{T}},$$

such that

3 $${\rm{Q}}=({\rm{\lambda }}+{\rm{\kappa }})\,{\rm{\Delta }}{\rm{T}}$$

It then becomes evident that N is a constant fraction of Q. If the radiative forcing Q decreases, the TOA radiative imbalance, N, and net OHU also decrease.

Alternatively, a hiatus can be forced by internal climate dynamics. In that case, during the hiatus Q keeps increasing while ΔT stalls. The increased radiative forcing during the hiatus period ΔQ then has to be balanced by in an increased ΔN;

4 $${\rm{\Delta }}{\rm{Q}}={\rm{\Delta }}{\rm{N}},$$

Alternatively a perturbation can be added to the forced response balance of Eq. 1, that compensates the radiatively forced temperature increase:

5 $$0={{\rm{\lambda }}}_{{\rm{N}}}\,{{\rm{T}}}_{{\rm{N}}}+{{\rm{N}}}_{{\rm{N}}},$$

where the subscript N denotes a natural fluctuation. Then it becomes clear that if T_N_ is negative (the negative of ΔT during a hiatus period), N_N_ has to be positive, i.e. the OHU and the TOA radiation imbalance become larger. This is the opposite relation between temperature variation and variation in N than in case of a radiatively forced hiatus. Such an opposite reaction in N also implies that Eq. 2 cannot hold for natural fluctuations, as Eq. 2 predicts a positive correlation between N and temperature fluctuations. As a result, the OHU efficiency κ has to change and has to become larger for a hiatus period and smaller for a positive natural temperature fluctuation. This can be explained by the fact that κ is essentially a diffusivity parameter, but that natural changes in OHU efficiency could be brought about by changes in deep convection and overturning circulation^17,18^, which are not well captured by a relation like Eq. 2.

Such anti-correlation between OHU, or N and GSMT has not been demonstrated to exist, neither in climate models, nor in observations. The implication then would be that the forcing-feedback-response energy balance model does not hold for dynamical fluctuations. This is especially puzzling as this model has recently been extended to account for (slow) changes in λ and κ that occur when the radiative forcing no longer increases and the planetary system approaches equilibrium^19,20^. If the transient behaviour in λ and κ is accounted for, an efficacy parameter, ε, can be defined, and the total temperature response can be split into a response to radiative forcing and a response to OHU forcing with the response to the latter forcing being larger by the factor ε^19^. This extended radiative-forcing-response model does not allow for decoupling of GMST variations from OHU variations, as it explicitly recognizes that all other forcing than radiative forcing must be moderated by OHU.

The lack of evidence for such a relation, both in observational studies^14,21^ and in analyses of unforced model simulations^22,23^, lead to the conjecture that the warming hiatus was a pure surface phenomenon due to a vertical redistribution of OHC only. Some support, however, for a relation between OHU/N and GMST has been found. For instance, the ORAS reanalysis of OHC shows a steep acceleration in OHC from year 2000 to 2010 relative to the decade before^24^, implying increased OHU during the hiatus period. Other OHC-data products, however, do not show such an acceleration^11^. A recent analysis claimed that reliable estimates of OHC since 1960 can now be made^25^. There still remains the issue that a reliable estimate of the relation between OHU and GMST is elusive as the length of the observational record is insufficient to separate different signals at different timescales. For this reason, analysing the relation in CMIP5 models is valuable.

Recent analyses of the relation between N at the TOA and GMST in climate models found a complex lead-lag relation between N and GMST at decadal timescales, peaking when GMST was leading with negative correlation, but with positive correlation when N was leading by more than a year^26^. Here, this analysis is extended by examining natural fluctuations between GMST and OHU, and by using forced simulations in which this relation might be affected by the forced response. By decomposing OHU in its various components, improved insight in the various feedbacks acting on OHU-forced temperature variations can be obtained, further elucidating the role of water vapour feedbacks (latent and sensible heat flux), Planck feedback (longwave radiation), and cloud and ice-albedo feedbacks (shortwave radiation). To isolate natural fluctuations in forced scenario runs performed by climate models, only single-model ensembles are considered from which the ensemble mean (forced) response is subtracted. It should be stressed that in this set-up only the mechanisms of how hiatus and surge events arise in climate models can be assessed and that the results do not need to be representative for the recent hiatus period. In particular, it was assessed that climate models have strong biases in simulating the ocean energy change associated with the El Niño-Southern Oscillation (ENSO), and that the subsequent area-averaged tropical Pacific OHC variability is greatly underestimated^27^. Nevertheless, understanding how hiatus events arise in climate models yields observable metrics that can verify/falsify whether the models behave in a similar way as the real world, and the qualitative relation between OHU and GMST could still be correct, despite biases like weaker GMST variance as observed, and as a result, weaker and less simulated hiatus periods than observed, and too weak OHU variations associated with ENSO.

## GMST and Heat Uptake in CMIP5 models

**Figure 1:**
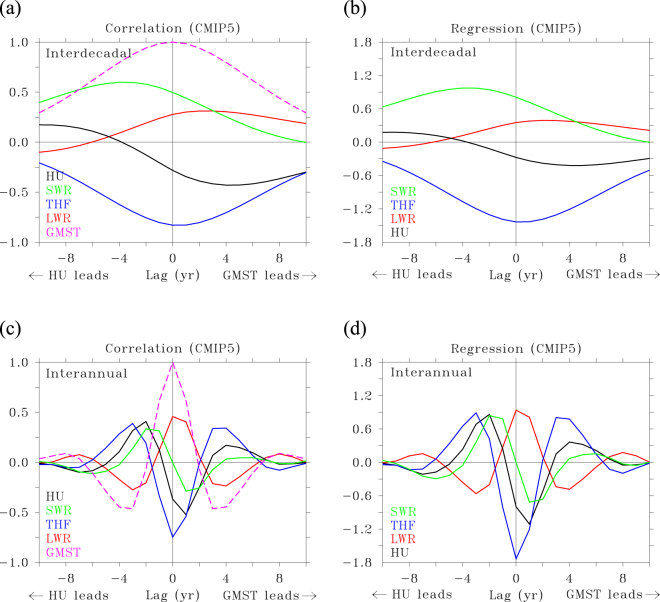
Relationship between GMST and HU in natural variability. (**a**–**d**) Lagged correlation (**a**) and lagged regression (**b**) of net heat uptake HU, net shortwave radiation (SWR), net longwave radiation (LWR) and turbulent (sensible plus latent) flux (THF; all positive downward) against GMST, based on decadal variations. Positive lags indicate that GMST leads; negative lags indicate that HU leads. Correlation is dimensionless. Regression values are in W m^−2^ K^−1^. Lagged correlation (**c**) and lagged regression (**d**) for inter-annual variations.

The general aspects of the relation between OHU and GMST in climate models is assessed by focusing on the ensemble mean response of multiple single-model ensembles, and looking for detailed lead-lag relationships between (O)HU and GMST. Because inter-annual and decadal variations might exhibit a different relation between OHU and GMST, the two timescales have been separated (Methods). Figure 1a shows the lagged correlation between all components of HU and GMST averaged over all realisations of a historical plus climate change simulation (RCP8.5) from six models participating in AR5 (Methods) for ***decadal*** timescales; Fig. 1b shows the lagged regression for decadal timescales. The number of models included in the analysis was constrained by the requirement that (1) at least three different realisations of the scenario were available, to allow calculation of an ensemble-mean response and anomalies from the ensemble-mean, and (2) all terms of the TOA and surface energy budget were available in the CMIP5 archive. In this, and in all following figures only natural, unforced, annual average variations are considered (see Methods).

In Fig. 1 HU is positive when the flux is downward. GMST and HU are anti-correlated at lag 0 (−0.3), peaking at lag 4 (−0.4; Fig. 1a) when HU lags GMST by 4 years. Correlations are significant when their absolute value is larger than 0.08 (Methods). At most leads and lags HU over land is small compared to HU over the ocean (Supplementary Fig. 1a), hence we neglect HU over land and equate it with OHU. Anti-correlation between OHU and GMST implies that the ocean releases heat to the atmosphere when GMST is positive, and vice versa. OHU and GMST anti-correlate, but with a certain phase lag and the correlation changes sign at lag −4 (Fig. 1a), implying that OHU and GMST are positively correlated when OHU leads by 4 years or more. This behaviour is qualitatively robust for all six models (Supplementary Fig. 1b). Consistent with the assumptions that both the heat capacity of the atmosphere and heat uptake/release by land and sea ice are negligible^6^, the lagged regression between OHU and GMST (Fig. 1b) is very similar to that between TOA radiation and GMST (Supplementary Fig. 1c). Based on the shift between natural variations in GMST and TOA radiation (Fig. 1b and Supplementary Fig. 1c) it was argued that the climate feedback arising from TOA radiation changes should read:

6 $${{\rm{Q}}}_{{\rm{C}}}=-\,{{\rm{\lambda }}}_{{\rm{F}}}\,{{\rm{T}}}_{{\rm{F}}}-{{\rm{\lambda }}}_{{\rm{N}}}{{\rm{e}}}^{-{\rm{i}}{\rm{\varphi }}}\,{{\rm{T}}}_{{\rm{N}}},$$

where Q_C_ is the climate feedback, T_F_ is the forced temperature response and T_N_ is a natural temperature variation. φ is the phase difference by which T_N_ leads outgoing TOA radiation, or in the present case, OHU. As can be seen in Fig. 1a,b OHU is phase shifted with respect to GMST and only weakly anti-correlated. When φ is exactly 90° averaged over the positive or negative phase (180°) of a harmonic oscillation in T_N_, the relation between OHU and GMST becomes zero^26^. Figure 1a,b features a smaller phase shift than 90° and also the oscillations in OHU and GMST are not sinusoidal, leading to a distinct correlation pattern as function of lead-lag. The lagged regression (λ_N_, see Fig. 1b) attains a maximum value of −0.5 W m^−2^ K^−1^, distinctly less than the estimated equilibrium climate feedback parameter^26^.

For ***inter-annual*** timescales the relation is different. Anti-correlation at lag 0 slightly increases compared to decadal timescales (−0.4), peaking at lag 1 (−0.5; Fig. 1c). For inter-annual variations, correlations are significant when their absolute value is larger than 0.039. At lag −2 and +4 there are now distinct positive maxima in correlation, indicating the relation is dominated by a 3-year timescale oscillation associated with ENSO, reflected in the autocorrelation of GMST. Beyond lags −8 and +8 the relation between HU and GMST becomes small and the autocorrelation drops to zero. The behaviour is again robust for all 6 models (Supplementary Fig. 1d). For inter-annual variations the climate feedback parameter is larger than for decadal variations: 1.0 W m^−2^ K^−1^ versus 0.5 W m^−2^ K^−1^ (Fig. 1b,d), implying twice as weak temperature variation for a given OHU anomaly compared to a decadal variation.

## Ocean forcing the atmosphere

A better understanding of how the phase shift between OHU and GMST arises is achieved by evaluating how the separate components of OHU relate to GMST. From Fig. 1a it can be seen that for ***decadal*** variations the surface turbulent heat fluxes (consisting of latent plus sensible heat) are always negatively correlated with GMST and this correlation peaks at zero lag with a value of −0.8. This anti-correlation is generally viewed as an indication that the ocean is forcing the atmosphere (heat release (uptake) during a surge (hiatus) period), while a positive correlation would indicate that the atmosphere is the forcing agent^28^. Longwave radiation behaves in the opposite sense, it is positively, but weaker correlated with GMST, peaking at lag 4 with a value of 0.25, and always damping GMST variations, transferring part of the anomalous heat released by turbulent fluxes back into the ocean. Although upward longwave radiation must increase (decrease) when GMST increases (decreases), being a positive function of temperature (i.e. εσT^4^, with ε the emissivity and σ the Stefan-Boltzmann constant), back radiation from an anomalous warm (cold) and moist (dry) atmosphere increases (decreases) even more. Turbulent fluxes show no lag with GMST, but longwave and shortwave radiation do show a lag. They are both positively correlated with GMST, so act as damping. While longwave radiation is lagging GMST, shortwave radiation is leading GMST. It peaks 4-years before GMST peaks (lag = −4), with a correlation of 0.6. Before the ocean releases heat to warm the atmosphere via turbulent fluxes, the ocean is warmed by larger amounts of solar radiation, and vice versa for heat uptake and cooling.

Also for ***inter-annual*** variations, turbulent fluxes anti-correlate with GMST and longwave radiation shows positive correlation with a small lag. The phase shift between shortwave radiation and GMST is larger, and from Fig. 1d it is unclear whether it is positively correlated and leading GMST, or negatively correlated and lagging GMST

## The trigger provided by changes in reflected solar radiation at the surface

To illustrate the effect of changes in reflected solar radiation on GMST, a negative GMST-anomaly (hiatus) is assumed while depicting the chain of processes leading to such an event. As a result, positive correlation and regression in Fig. 1 implies weaker than normal OHU, negative correlation and regression imply stronger than normal OHU.

**Figure 2:**
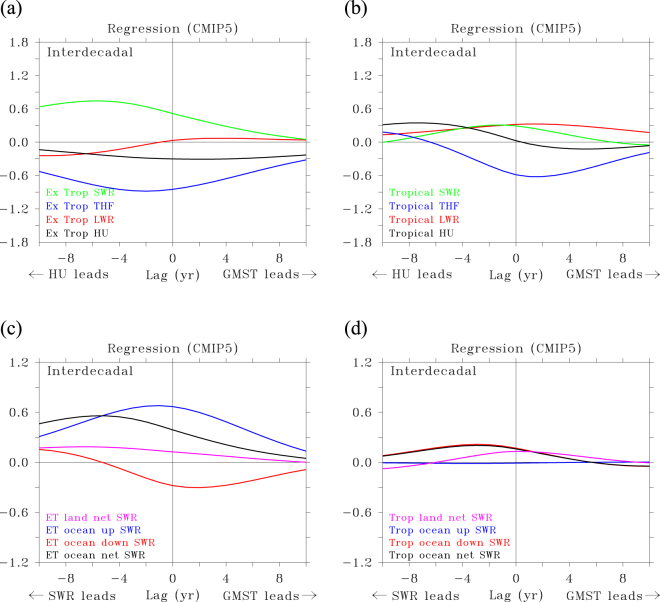
Relationship between GMST and HU/SWR in decadal variations for different areas. (**a**–**d**) Lagged regression (**a**,**b**) of net heat uptake HU and net shortwave radiation (SWR) (**c**,**d**), against GMST. (**a**,**b**) HU split between tropics (30°S and 30°N) and extra-tropics such that both panels sum to Fig. 1. (**c**,**d**) SWR split between tropics and extra-tropics, ocean and land, and upward and downward shortwave radiation such that the black and purple lines in both panels sum to the green line in Fig. 1. Regression values are in W m^−2^ K^−1^.

From Fig. 1a,b one can infer that for ***decadal*** variations, before a hiatus arises net OHU is negative by increased reflection of solar radiation. Part of the resulting cooling is passed to the atmosphere through increased net uptake by turbulent fluxes. Since turbulent fluxes primarily ***release*** heat to the atmosphere, net uptake must result from releasing ***less*** heat to the atmosphere. This means that the atmosphere cools and becomes drier. These changes start at least 10 years before the hiatus peaks and become stronger over time, until 4 years before the minimum in GMST occurs (lag = −4). It was previously suggested that such changes in reflected solar radiation result from stochastic atmospheric forcing, that is, changes in cloud cover^26^. When examining the energy budget at the surface it becomes clear that the changes in reflected solar radiation have a different cause. Figure 2a,b show that reduced absorption of solar radiation primarily occurs in the extra-tropics. There, reduced absorption is dominated by increased upwelling shortwave radiation (Fig. 2c). For lead times larger than 6 years increased upwelling shortwave radiation is associated with decreased down-welling radiation, which means that the increased upwelling is due to enhanced reflection of solar radiation. For decreasing lead time both upwelling and down-welling shortwave radiation increase, so the initial increased reflection of solar radiation is further enhanced by increased down-welling. This strongly suggests that increased sea-ice cover reflects more solar radiation at large lead times, after which increased reflection is further enhanced by increased down-welling of shortwave radiation due to decreased reflection by cloud cover, because the atmosphere becomes drier as sea ice increases.

**Figure 3:**
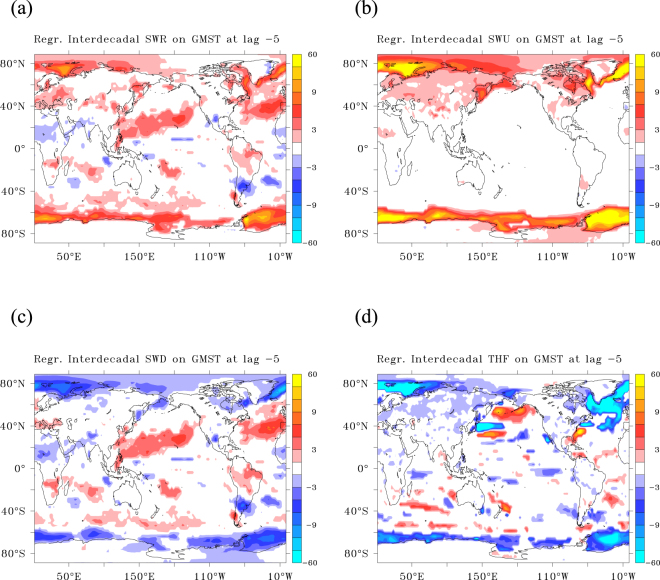
The SWR and THF patterns preceding a decadal GMST anomaly. (**a**–**d**) Regression patterns at lag −5, leading GMST by 5 years. Net shortwave radiation leading GMST by 5 years (**a**); same for upwelling shortwave radiation (**b**); downwelling shortwave radiation (**c**); Turbulent fluxes (**d**). All regression patterns are in (W m^−2^ K^−1^). In (**a**,**d**) colour intervals are 2 W m^−2^ K^−1^, but the first and last intervals are −60 to −13 and 13 to 60 W m^−2^ K^−1^. Only significant values using 95% confidence intervals are shown. This figure has been created with the free Ferret software package developed by the National Oceanic and Atmospheric Administration (NOAA) and available from www.ferret.noaa.gov.

This mechanism is further illustrated by examining the regression patterns for shortwave radiation at the surface and for the turbulent fluxes 5 years before the hiatus peaks (Fig. 3). At this time, increased reflection of solar radiation cools most of the subpolar latitudes which feature sea-ice and snow cover in winter (Fig. 3b). Decreased down-welling shortwave radiation features tilted bands of 20° width in the subtropical to mid-latitude North Pacific and Atlantic, and weaker zonal bands in the Southern Hemisphere mid-latitudes (Fig. 3c). Increased reflection at the surface occurs primarily ***poleward*** of 50° with hot spots over deep convection sites (Fig. 3b). There, increased reflection is partly counteracted by increased down-welling shortwave radiation, that is reduced reflection by clouds (Fig. 3c). From Fig. 3c it is also inferred that ***equatorward*** of 50°, increased reflection by clouds occurs, reducing down-welling shortwave radiation at lower latitudes.

At subpolar latitudes, the turbulent fluxes release less heat to the atmosphere, causing net ocean heat uptake, with hotspots at the deep convection sites where they anti-correlate with increased reflection of shortwave radiation. Reduced heat release by turbulent fluxes and increased sea-ice cover are indicative of weakened convective mixing and a weakening of the sinking branch of the overturning circulation^29^.

Interestingly, distinct dipole patterns in turbulent fluxes are visible between 30° and 40°N, where the jet stream leaves the continent, affecting the storm genesis areas over the North Atlantic and North Pacific. Similar patterns are also visible at 10 years lead time (Supplementary Fig. 2).

## The short lead-time response dominated by the tropics

**Figure 4:**
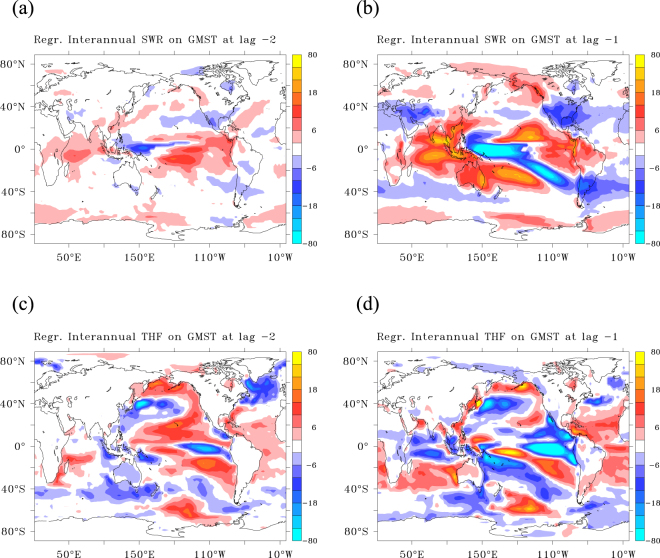
The SWR and THF patterns preceding an inter-annual GMST anomaly. (**a**–**d**) Regression patterns at different lags. Net shortwave radiation leading GMST by 2 years (**a**), and 1 year (**b**). Turbulent flux leading GMST by 2 years (**c**), and 1 years (**d**). All regression patterns are in (W m^−2^ K^−1^). In (**a**,**d**) colour intervals are 4 W m^−2^ K^−1^, but the first and last intervals are −80 to −26 and 26 to 80 W m^−2^ K^−1^. Only significant values using 95% confidence intervals are shown. This figure has been created with the free Ferret software package developed by the National Oceanic and Atmospheric Administration (NOAA) and available from www.ferret.noaa.gov.

For ***inter-annual*** variations a trigger in the form of changes in energy budget is less apparent. Shortwave radiation and turbulent fluxes at 1 and 2 year leads are dominated by moving patterns in the tropical Pacific and changes in absorbed solar radiation are associated with changes in cloud reflection (Fig. 4), dominated by changes in down-welling shortwave radiation. These moving patterns are in stark contrast with the stationary patterns at subpolar latitudes that lead decadal variations in GMST. The absorbed shortwave radiation anomalies in the tropics reveal that the warming pattern associated with reduced cloud cover propagates eastward, while the cooling patterns, associated with enhanced cloud cover, move westward. The changes in OHU reflect the ENSO-cycle and cannot be regarded as independent precursors. Remarkably, a stronger than normal heat release in the subpolar North Atlantic by turbulent fluxes at 2-year lead-time (Fig. 4c) exists, which at first sight seems to play a similar precursor role as for decadal variations in GMST. However, there is no associated signal in upwelling shortwave radiation and the signal disappears at 1 year lead time. At zero lag it shows-up again, but now with the opposite sign (Supplementary Fig. 3), and it appears here as an in-phase response to ENSO instead of a trigger preceding it.

**Figure 5:**
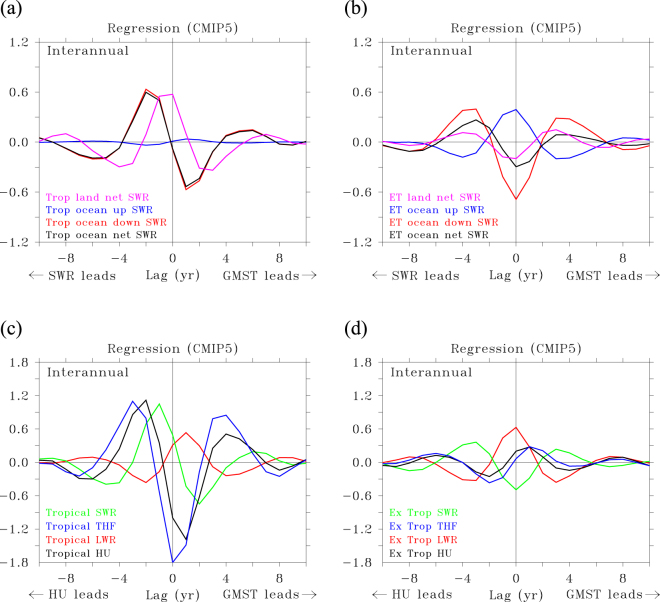
Relationship between GMST and HU/SWR in inter-annual variations for different areas. (**a**–**d**) Lagged regression (**a**,**b**) of net shortwave radiation (SWR) and of net heat uptake HU (**c**,**d**), against GMST. (**a**,**b**) SWR split between tropics (30°S and 30°N) and extra-tropics, ocean and land, and upward and downward shortwave radiation such that the black and purple lines in both panels sum to the green line in Fig. 1; HU split between tropics and extra-tropics such that both panels sum to Fig. 1. (**c**,**d**) Regression values are in W m^−2^ K^−1^.

At lead-times of 2 to 4 years, reflected solar radiation is enhanced, but it decreases at shorter lead times, to become negative after the peak of an inter-annual hiatus (Fig. 5,a,b). For inter-annual variations, reflected solar radiation mainly decreases through diminished reflection by clouds (less down-welling shortwave radiation), especially in the tropics. In the extra-tropics, changes in upward shortwave radiation damp the changes in down-welling shortwave radiation. When it is cold an dry, less shortwave radiation is reflected by clouds (more down-welling shortwave radiation), but more short-wave radiation is reflected by sea-ice (more upwelling shortwave radiation), and vice versa when it is warm (Fig. 5b). In the extra-tropics, the shortwave radiation changes are in phase with GMST, implying a fast response to ENSO, while in the tropics they show a phase lag with GMST, being part of the ENSO-cycle that drives GMST variations.

Between 4 years and 1 year before the hiatus peaks, cloud cover, following the earlier increase in latent heat release, increasingly reflects solar radiation in the tropics (Fig. 5c). When cloud cover increases, heat release by the turbulent fluxes quickly drops, resulting in a strong minimum in heat release and a maximum in net heat uptake just after the peak of the hiatus. At short lead-times, the tropical oceans become the dominant sink of heat for the atmosphere, with OHU peaking 1 year after the hiatus through strongly decreased latent and sensible heat release, while the extra-tropics show the opposite behaviour, but with weaker amplitude (Fig. 5c,d). At the peak of an inter-annual hiatus, the pattern of OHU is dominated by heat uptake in the tropical Pacific (Supplementary Fig. 3), with large, negative values in the tropical Pacific (heat uptake) and large positive values in the northwest Pacific and the eastern part of the Indian Ocean (heat release). Surprisingly, the regression patterns of TOA and OHU are anti-correlated, especially in the tropical Pacific (Supplementary Fig. 4), although their global averaged values are positively correlated. This reflects how much the local patterns are determined by warm and cold air anomalies losing/gaining equal amounts of heat at the TOA and at the surface.

## The signature in SAT and SST

**Figure 6:**
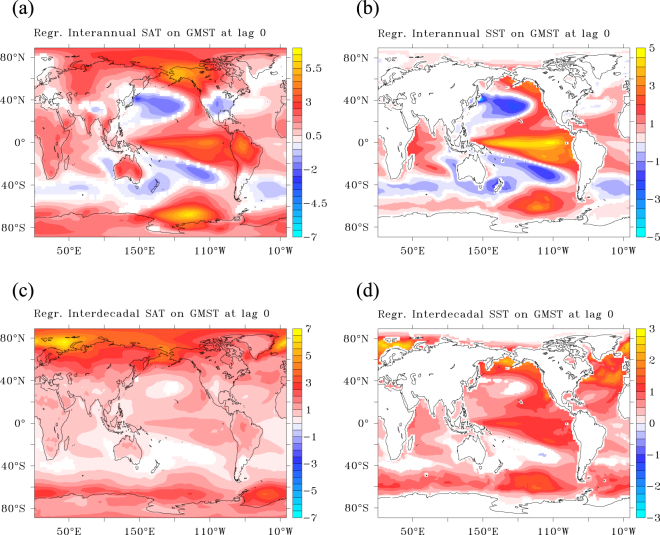
The temperature patterns during an inter-annual and decadal GMST anomaly. (**a**–**d**) Regression on GMST at lag 0. The regression of surface air temperature (K K^−1^) (**a**,**c**), and of sea surface temperature (K K^−1^) (**b**,**d**). Only significant values using 95% confidence intervals are shown. This figure has been created with the free Ferret software package developed by the National Oceanic and Atmospheric Administration (NOAA) and available from www.ferret.noaa.gov.

For ***inter-annual*** variations the response in SAT and sea-surface temperature (SST) reproduce the ENSO-Pacific Decadal Oscillation (PDO) signal that was also dominant in the observed, recent decadal hiatus^30–32^ (Fig. 6a,b). In addition, SAT also shows large regression values in polar and subpolar oceans, which are weaker in SST. The decoupling of SST and SAT over the ocean can only arise when SAT anomalies are due to anomalies in sea-ice cover, over land they must be amplified by changes in snow cover.

For ***decadal*** variations the response in SST, and to a lesser extent in SAT, still contains the signal of the ENSO-PDO. However, much more pronounced is a polar-amplified global warming signal, peaking over the areas of ocean deep convection (Fig. 6c,d). This signal is clearly distinct from the pattern associated with the observed recent hiatus^24,30,31^. The fact that the amplitude of the observed climate hiatus was outside the 95% band of decadal internal variability in GMST in the CMIP5 ensemble^33^, and that prescribing the surface characteristics of this variability did reproduce the observed signal in GMST^30,31,34^ in CMIP5 models, strongly suggests that too weak decadal variability in simulated GMST in CMIP5 is associated with too weak decadal variability in the (tropical) Pacific, hence the overly strong signature of subpolar latitudes in decadal GMST variations in CMIP5. However, it should be noted that despite this bias, the subpolar precursors in form of changes in sea-ice cover and ocean deep convection appear robust and also apply when GMST is limited to tropical latitudes (Supplementary Fig. 5). Also recent observational analyses confirm such links between subpolar and tropical latitudes in the climate hiatus^3,35^. At 10-year lead times, the signature of SAT and SST contain strong signals associated with changes in sea-ice in the Norwegian and Barents Sea, indicative of changes in inflow into the Arctic by the North Atlantic Drift – Norwegian Current, but also the signature of the opposite phase of the PDO is present (Supplementary Fig. 6), suggesting that both changes in AMOC and PDO might be precursors of a decadal climate hiatus.

## Conclusion and Outlook

In climate models the correlation between OHU (or N) and GMST is multi-signed, depending on the lead-lag. The maximum regression factor between the two is about twice as weak as the equilibrium climate feedback factor. This can be reconciled by noting that the OHU feedback factor must and does equal the equilibrium climate feedback factor divided by the heat uptake efficacy^19^, ε, which here is estimated to be about 2 for decadal variations. This implies that a stall in temperature increase can arise when the increase in radiative forcing is compensated by a twice as weak increase in OHU or TOA radiation imbalance:

7 $$0={{\rm{\lambda }}}_{{\rm{eq}}}\,{\rm{\Delta }}{\rm{T}}={\rm{\Delta }}{\rm{Q}}-{\rm{\varepsilon }}\,{\rm{\Delta }}{\rm{N}}$$

Such an increase in OHU or N for the recent hiatus has not been detected in the observations, but it should be noted that the implied increase of order 0.1 W m^−2^ is too small to detect, given the uncertainty in the measurements.

The climate models agree in the temperature signal of a hiatus period, but the tropical Pacific appears too weak in decadal variations. It has been argued that biases in mean convective activity in the eastern Pacific lead to incorrect changes in low level clouds and as a result, in TOA radiation and surface fluxes. Associated with this bias the ENSO signal is too weak in 0–700 m OHC^27^. It can be anticipated that without these biases, variations in OHC and OHU with ENSO would become larger, leading to stronger variations in ocean heat uptake efficiency, which indeed appear stronger in observations than in CMIP5 models^33^. Because the variations in OHC associated with ENSO in CMIP5 appear qualitatively correct, but quantitatively too weak^27^, the qualitative relation between OHU and GMST discussed here is likely much less affected by these biases than its magnitude, and would appear even more pronounced if these biases were remedied.

## Methods

### CMIP5 simulations

The models used were CanESM2 (5 members), CCSM4 (6 members), CNRM-CM5 (5 members), CSIRO-Mk3-6-0 (10 members), HadGEM2-ES (3 members), IPSL-CM5A-LR (4 members). Natural variations were derived from the historical and RCP8.5 simulations, which have been concatenated, giving 240 years per ensemble member. The RCP8.5 scenario was chosen as more models runs were available for this scenario, and by focusing on natural variations only, the choice of which scenario is less important. To isolate internal variations in each model, the ensemble-mean of each variable (SAT and all components of HU and TOA radiation) was estimated and subtracted from each individual ensemble member. To isolate decadal and longer timescales in these variations, the resulting timeseries were filtered with a 13-year Welch filter and thereafter linearly detrended, and any remaining average value was subtracted to obtain timeseries of exact zero-mean value and zero-trend. To isolate inter-annual timescales, the resulting timeseries were filtered with a 3-year Welch filter from which the timeseries filtered with a 13-year Welch filter was subtracted. In the multi-model-mean ensemble each model was given the same weight, irrespective of the number of ensemble members available. The total length of all timeseries became 33 × 240 years, implying for inter-annual variations a lower bound of 2638 degrees of freedom for significance tests, and for decadal variations a lower bound of 607 degrees of freedom (we are not interested to determine the exact significance bound which has to be estimated by a suite of Monte Carlo simulations, as all relevant results are clearly significant and supported by physical understanding). Then, correlations are significant at a 95% confidence level when they exceed 0.08, or −0.08, for decadal variations and 0.039, or −0.039, for inter-annual variations according to a two-tailed Student’s T-test.

### Lagged correlation and regression

Correlation describes the ***degree*** to which two variables, here HU and GMST, are related. It is measured by the covariance of the two, divided by the standard deviation of each of the two variables. It is dimensionless and varies between −1 and 1. Regression describes the amount by which one variable (HU) varies, given the observed variation in another variable. If *r* is the correlation coefficient that the regression value equals r times the standard deviation in variable *Y* (HU), divided by the standard deviation in variable *X* (GMST). In this case it has the dimension W m^−2^ K^−1^. By shifting timeseries it can be assessed to what extent the two variables are related when one variable leads the other. This gives information on possible causal relations. The figures shown here are expressed as lags in GMST. A negative lag then implies HU is leading in time with 1–10 years, depending on the amount of lag, while a positive lag implies GMST is leading in time. This can be applied to both correlation and regression. By showing lagged correlation and regression plots it can be assessed how the relation between HU and GMST changes when the lead-time between the two variables changes, that is, when the relation changes between forcing and response.

## Electronic supplementary material

Supplementary Information
