# Supplementary material for: The relation between natural variations in ocean heat uptake and global mean surface temperature anomalies in CMIP5
Source: Sci Rep. 2018 May 9;8:7402. doi: 10.1038/s41598-018-25342-7 (PMC5943251; doi:10.1038/s41598-018-25342-7)
Supplement: Supplementary file 1 — Supplementary Information [file 41598_2018_25342_MOESM1_ESM.pdf]

# The relation between natural variations in ocean heat uptake and global mean surface temperature anomalies in CMIP5.

**Sybren Drijfhout, University of Southampton, UK & Royal Netherlands Meteorological Institute**

**Supplementary Information:**

**Supplementary Data**

**Supplementary Figure 1:** Lagged correlations and regressions of heat uptake and TOA radiation against GMST.

**Supplementary Figure 2:** The SWR and THF patterns preceding a decadal GMST anomaly at 10-year lead-time.

**Supplementary Figure 3:** The heat uptake patterns during an inter-annual GMST anomaly.

**Supplementary Figure 4:** The heat uptake and TOA radiation pattern during an inter-annual GMST anomaly.

**Supplementary Figure 5:** The SWR and THF patterns preceding a decadal T(ropical)MST anomaly at 10-year lead-time.

**Supplementary Figure 6:** The temperature patterns preceding a decadal GMST anomaly at 10-year lead time.

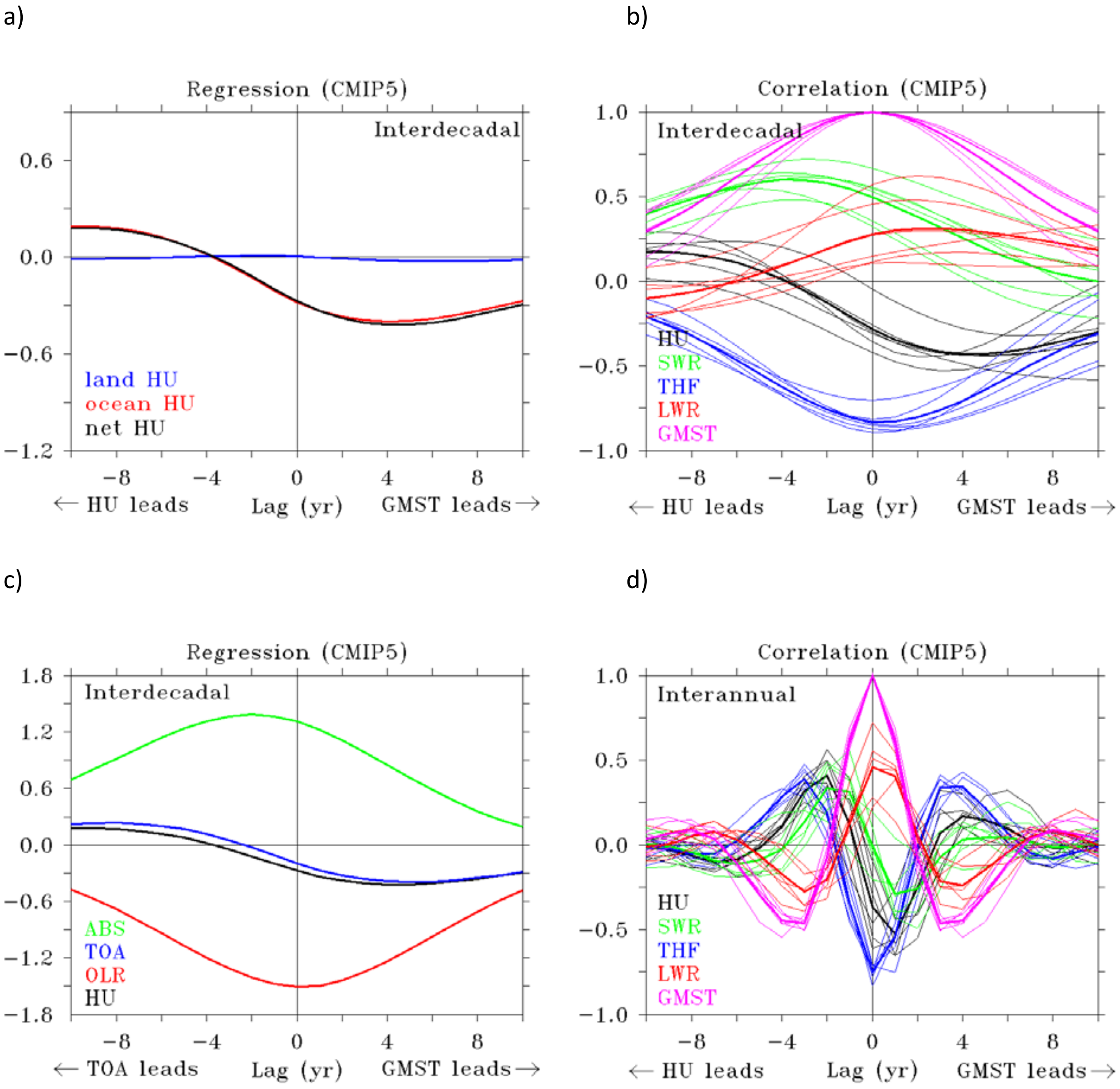


**Supplementary Figure 1. Lagged correlations and regressions of HU and TOA radiation against GMST.** a) Net heat uptake (HU) decomposed into heat uptake by land and ocean for decadal variations; b) Lagged correlation of net heat uptake HU, net shortwave radiation (SWR), net longwave radiation (LWR) and turbulent (sensible plus latent) flux against GMST. Multi-model-mean of CMIP5 models (thick curve), individual models thin curve (decadal variations); c) Lagged regression of net TOA radiation, decomposed into absorbed solar radiation (ASR) and minus outgoing longwave radiation (OLR) and net heat uptake HU (all positive downward) against GMST (decadal variations); d) Similar to panel b for inter-annual variations. Correlation is dimensionless. Regression values are in W $m^{-2}$ $K^{-1}$.

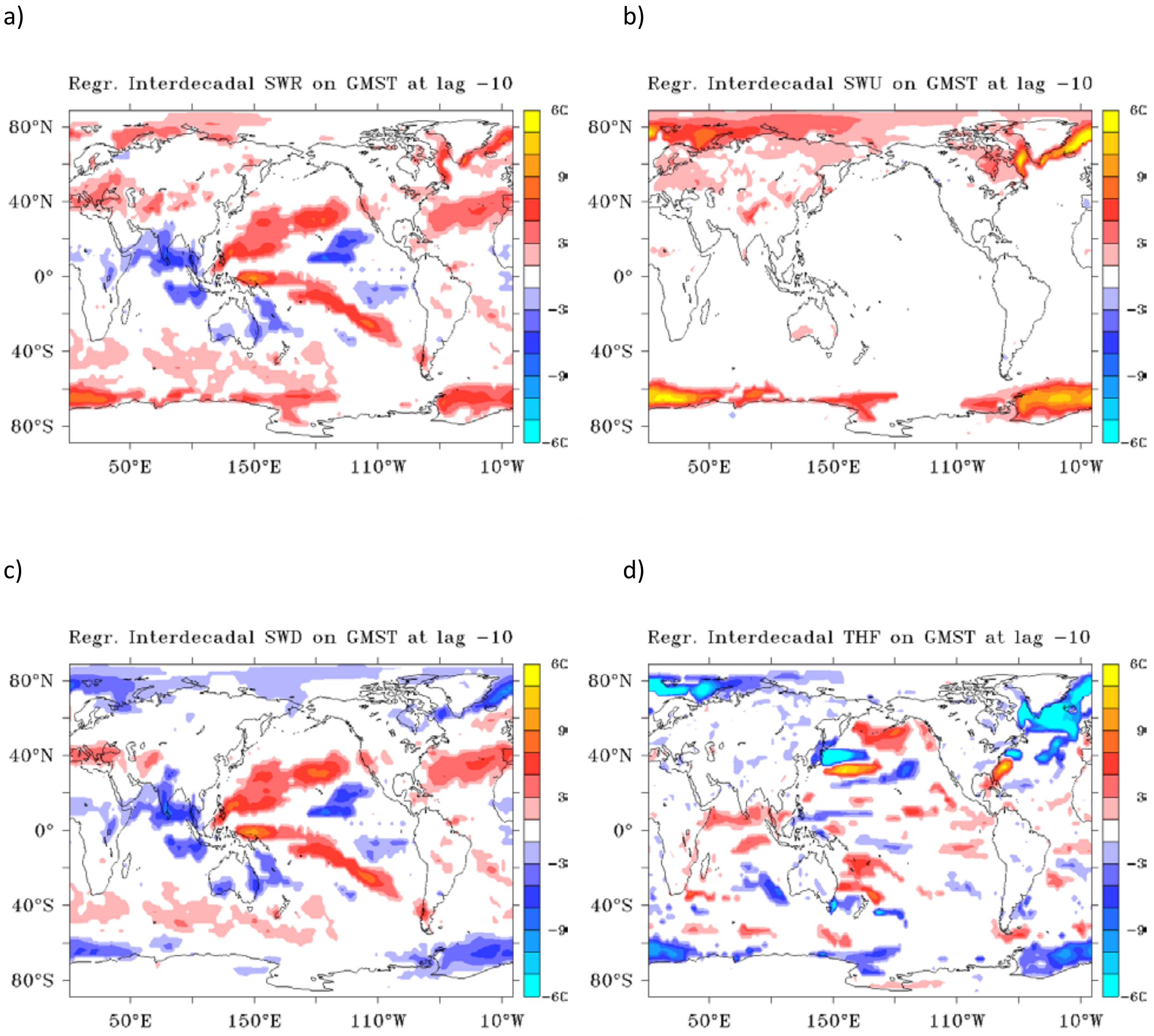


**Supplementary Figure 2. The SWR and THF patterns preceding a decadal GMST anomaly. a-d,** Regression patterns at lag -10. Net shortwave radiation leading GMST by 10 years **(a)**; same for upwelling shortwave radiation **(b)**; down-welling shortwave radiation **(c);** Turbulent fluxes (**d**)**.** All regression patterns are in (W $m^{-2}$ $K^{-1}$). In (**a,d**) colour intervals are 2 W $m^{-2}$ $K^{-1}$, but the first and last intervals are -60 to -13 and 13 to 60 W $m^{-2}$ $K^{-1}$. Only significant values using 95% confidence intervals are shown. This figure has been created with the free Ferret software package developed by the National Oceanic and Atmospheric Administration (NOAA) and available from www.ferret.noaa.gov.

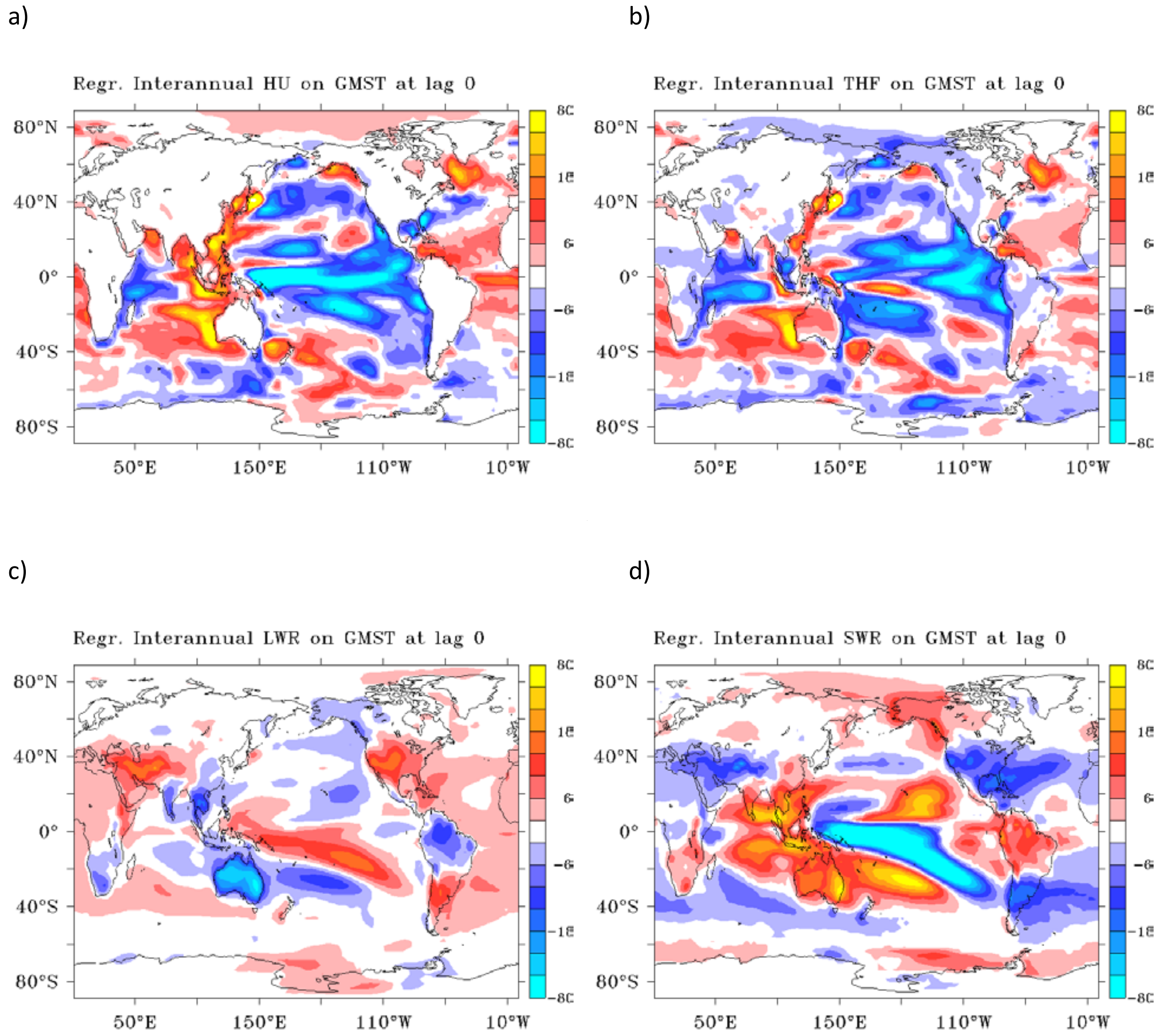


**Supplementary Figure 3. The HU patterns during an inter-annual GMST anomaly. a-d,** net heat uptake (W m$^{-2}$ K$^{-1}$) (**a**), turbulent flux (W m$^{-2}$ K$^{-1}$) (**b**), net longwave radiation (W m$^{-2}$ K$^{-1}$) (**c**), net shortwave radiation (**d**). In (**a,d**) colour intervals are 4 W m$^{-2}$ K$^{-1}$, but the first and last intervals are -80 to -26 and 26 to 80 W m$^{-2}$ K$^{-1}$. Only significant values using 95% confidence intervals are shown. This figure has been created with the free Ferret software package developed by the National Oceanic and Atmospheric Administration (NOAA) and available from www.ferret.noaa.gov.

a) b)

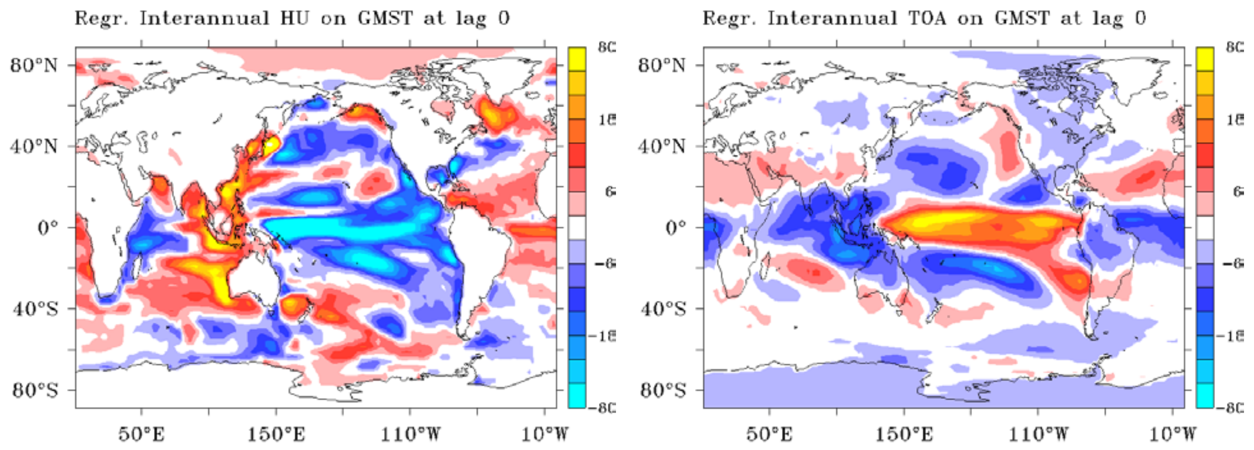


**Supplementary Figure 4. The HU and TOA pattern during an inter-annual GMST anomaly. a-b,** Regression on GMST at lag 0. The regression of net heat uptake (W $m^{-2}$ $K^{-1}$) (**a**), and net TOA radiation (W $m^{-2}$ $K^{-1}$) (**b**). Colour intervals are 4 W $m^{-2}$ $K^{-1}$, but the first and last intervals are -80 to -26 and 26 to 80 W $m^{-2}$ $K^{-1}$. Only significant values using 95% confidence intervals are shown. This figure has been created with the free Ferret software package developed by the National Oceanic and Atmospheric Administration (NOAA) and available from www.ferret.noaa.gov.

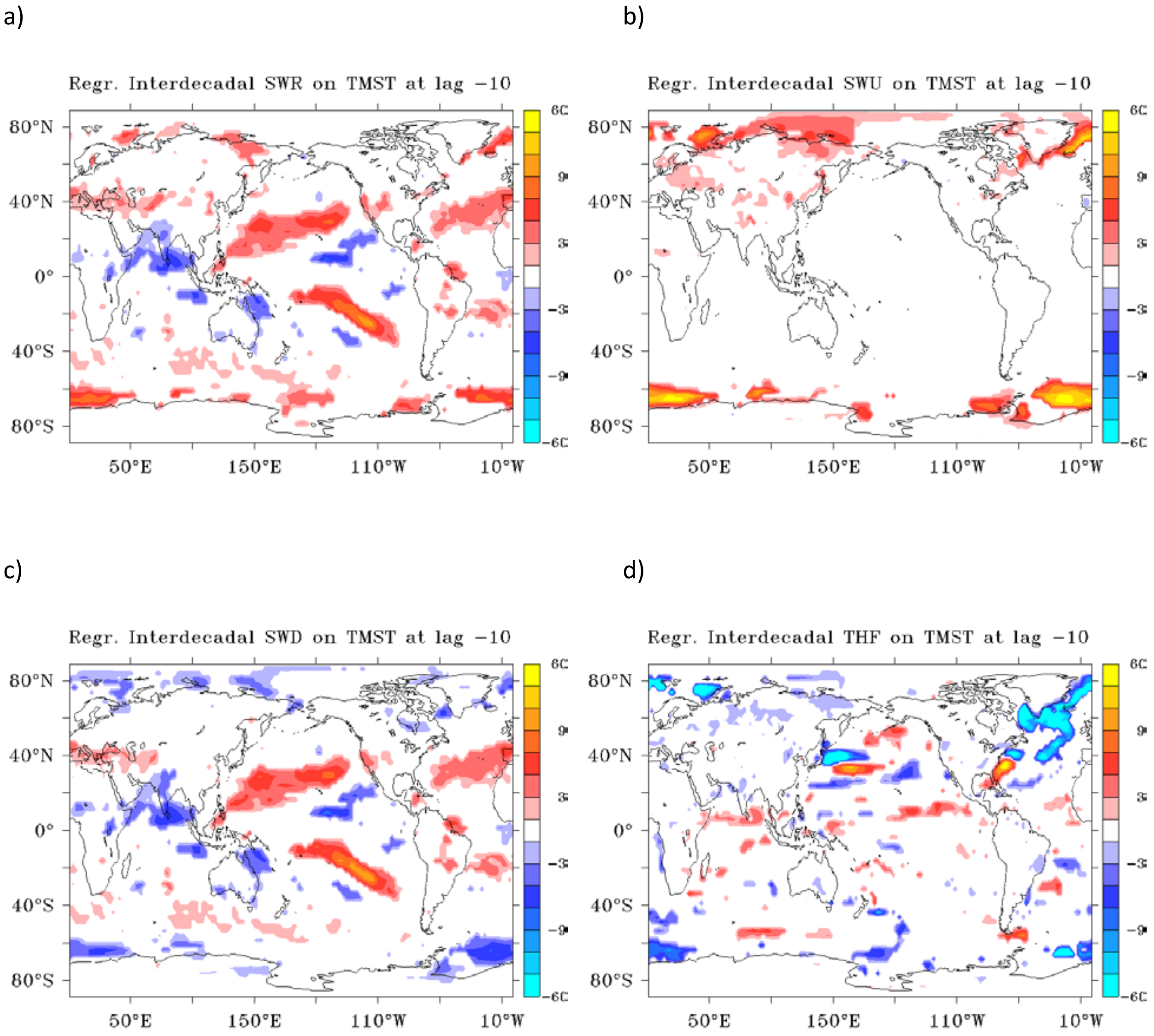


**Supplementary Figure 5. The SWR and THF patterns preceding a decadal TMST anomaly. a-d,** Regression patterns at lag -10. Net shortwave radiation leading T(ropical)MST by 10 years **(a)**; same for upwelling shortwave radiation **(b)**; down-welling shortwave radiation **(c);** Turbulent fluxes **(d).** All regression patterns are in (W $m^{-2}$ $K^{-1}$). In (**a,d**) colour intervals are 2 W $m^{-2}$ $K^{-1}$, but the first and last intervals are -60 to -13 and 13 to 60 W $m^{-2}$ $K^{-1}$. Only significant values using 95% confidence intervals are shown. This figure has been created with the free Ferret software package developed by the National Oceanic and Atmospheric Administration (NOAA) and available from www.ferret.noaa.gov.

a) b)

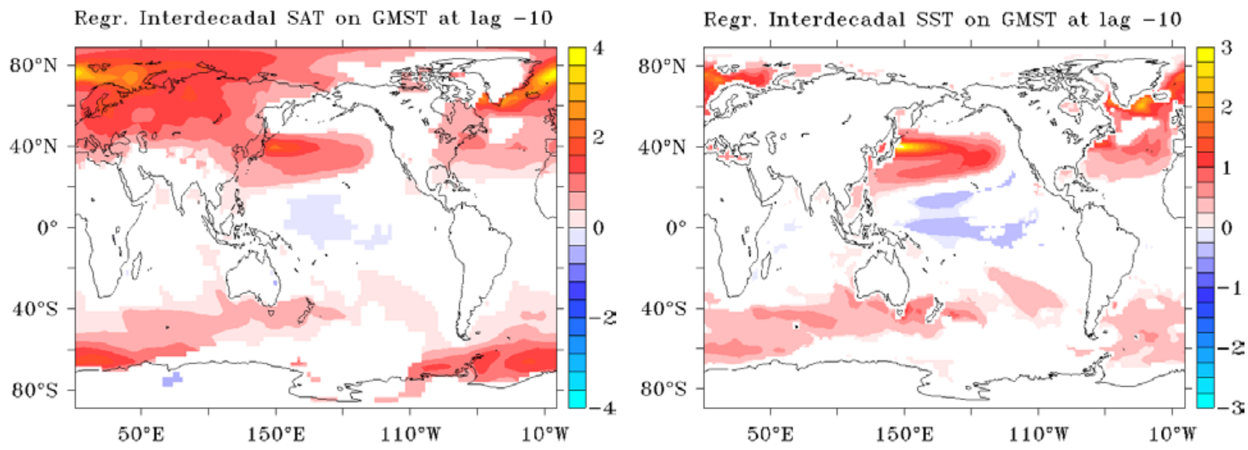


**Supplementary Figure 6. The temperature patterns preceding a decadal GMST anomaly. a-b,** Regression on GMST at lag -10. The regression of surface air temperature (K $K^{-1}$) (**a**), and of sea surface temperature (K $K^{-1}$) (**b**). Only significant values using 95% confidence intervals are shown. This figure has been created with the free Ferret software package developed by the National Oceanic and Atmospheric Administration (NOAA) and available from www.ferret.noaa.gov.
